# Response to Simon *et al.*,

**DOI:** 10.1186/s40478-017-0434-8

**Published:** 2017-04-29

**Authors:** Wei Wei, Michael J. Keogh, James W. Ironside, Patrick F. Chinnery

**Affiliations:** 1 Department of Clinical Neurosciences and MRC Mitochondrial Biology Unit, University of Cambridge, Cambridge, UK; 2 Centre for Clinical Brain Sciences, University of Edinburgh, Edinburgh, UK

## Please see the related Research article (10.1186/s40478-016-0404-6) and the related Letter to the Editor (10.1186/s40478-017-0433-9)

As Simon *et al.*, note, our study [1] was not designed to study low levels of heteroplasmic mitochondrial DNA (mtDNA) variants within specific cell types (such as in the *Substantia nigra*). In addition, Parkinson’s disease comprised only 3.2% of our cohort of 1363 *post mortem* brains [1]. We agree, therefore, that our findings do not directly contradict their published observations [2–4]. However, we did show that heteroplasmic mtDNA mutations are a common finding in the human brain [1]. Both the mean number of heteroplasmic single nucleotide variants (SNVs, with a variant allele frequency, VAF >10%), and the mean percentage level of mtDNA heteroplasmy in several neurodegenerative diseases (Alzheimer’s disease, frontotemporal dementia-amyotrophic lateral sclerosis, Creutzfeldt-Jakob disease, and Dementia with Lewy bodies–Parkinson’s disease [DLB-PD]) were no different to age-matched controls (Fig. 3 in Wei *et al*. [1],). In addition, both the mean number of heteroplasmic variants and the mean level of mtDNA heteroplasmy were not associated with age at death in the neurodegenerative disease cases, nor in controls. Consistent with other studies, many (if not all) of these variants are likely to have been inherited rather than acquired as somatic mutations during life [5], and are therefore highly likely to be present within the *Substantia nigra,* despite not being directly sampled. If the mtDNA variants we detected were contributing to the pathogenesis of neurodegeneration, we would have expected to see a difference between diseases cases and controls. If mtDNA mutations are contributing to cell loss, then one might even expect to see a reduction in the mutation burden in affected tissue - but this was not the case in our study (including DLB-PD, Fig. 3 & Supplementary Fig. 12 in Wei et al. [1],), and it was not the case in several studies of Simon *et al.* [2–4].

As Simon *et al.*, point out, they have shown that neurons containing mtDNA mutations are present in early and pre-clinical Lewy body disorders [2], thus demonstrating that the presence of a mtDNA mutation need not necessarily cause immediate neuronal loss. Any one individual mutation could have been inherited [5] (and been present from birth at low levels), particularly if it is shared by several cells. Given the inherent difficulties in studying post mortem tissue, the only way of definitively solving this issue will be through direct experimentation in a relevant animal or cell model of disease. For Parkinson’s disease, we need to know whether the introduction of mtDNA mutations actually causes neurodegeneration in the *Substantia nigra*, and is not just an innocent bystander in a toxic cellular environment at a particular phase of the disorder.
